# Occupational burnout and lifestyle in Kazakhstan cardiologists

**DOI:** 10.1186/s13690-019-0345-1

**Published:** 2019-04-10

**Authors:** Denis Vinnikov, Anar Dushpanova, Almat Kodasbaev, Zhanna Romanova, Aizhan Almukhanova, Zhangir Tulekov, Esbol Toleu, Gainel Ussatayeva

**Affiliations:** 1 Al-Fabari Kazakh National University, al-Farabi avenue 71, Almaty, Kazakhstan 050040; 2 Biological institute, National Research Tomsk State University, Tomsk, Russian Federation 634050; 3 City Cardiological Centre, Tolebi 93, Almaty, Kazakhstan 050000; 4 Asfendiyarov Kazakh National Medical University, Tolebi 94, Almaty, Kazakhstan 050000; 5 KSPH Kazakhstan Medical University, Utepova 19a, Almaty, Kazakhstan 050060

**Keywords:** Smoking, Alcohol, Fatigue, FSS

## Abstract

**Background:**

No data exist in the published literature on burnout in physicians from Central Asia. The aim of this analysis was to assess burnout prevalence in doctors and nurses of a cardiological hospital in Almaty, Kazakhstan and ascertain whether smoking, alcohol and physical activity may predict job-associated burnout.

**Methods:**

The staff of the City Cardiological Centre of Almaty (*N* = 259, 82% females) filled in the questionnaire with the questions on demographics, lifestyle, including smoking, alcohol and physical activity, as well as fatigue (using Fatigue Severity Scale (FSS)) and burnout using Maslach Burnout Inventory (MBI) Human Services Survey for Medical Personnel. We compared the scores of emotional exhaustion (EE), depersonalization (DP) and personal accomplishment (PA) between doctors and nurses.

**Results:**

We found significant differences in the smoking prevalence, alcohol use and regular physical activity, but no mean scores of burnout between men and women. High DP was prevalent in 52% doctors and 45% nurses, whereas high EE was found in 32 and 26% and PA in 16 and 32%, accordingly. In contrast with EE and DP, PA score was greater in nurses (median 38, interquartile range (IQR) 17) compared to doctors (median 41, IQR 9). Age, sex, work duration, smoking or physical activity could not predict higher burnout, whereas FSS score was associated with higher burnout of all dimensions (*p* < 0.05), and never-alcohol could predict higher PA burnout (p < 0.05).

**Conclusions:**

In Kazakhstan cardiologists, high prevalence of DP burnout should target specific preventive strategies and the association of alcohol use with PA needs further deeper insight.

## Background

Occupational burnout is usually a stress-induced gradual reduction in satisfaction from work, work efficiency and feeling of reward as a consequence of prolonged stress, emotional and physical exhaustion. Because cynicism resulting from dissatisfaction and dropping professional accomplishment are now considered components of burnout, its three dimensions have been proposed by Maslach and Jackson [1] and widely used all over the world to quantify burnout in various occupations. They were derived firstly from human services, but with evolving data from research, it has become clear that these three scales of burnout are present and reported from almost all occupations. With twelve described stages of burnout [2], many of them may combine and accelerate destructive behavior and serious damage to the individual professional reputation and the institution rating. Quickly progressing and unaddressed burnout may even trigger suicidal behavior, especially in medicals [3].

In medical professionals, emotional and physical aspects of burnout along with impaired quality of life have been studied quite extensively [3]. The American College of Surgeons has undertaken probably the largest survey in physicians, in which almost 8000 members responded with the overall burnout prevalence 40% [4]. With almost every other physician affected, there is a notable difference in the prevalence among specialties, emergency medicine taking the lead and staff in preventive/occupational medicine least affected [5], whereas in all specialties the prevalence tends to grow in recent years. Besides, nurses may show even higher rates of burnout compared to doctors [6]. Various interventions have been proposed to combat burnout in physicians, and a systematic review of interventions showed that “both individual-focused and structural or organisational strategies can result in clinically meaningful reductions in burnout among physicians” [7].

No data exist in the published literature on burnout in physicians from Central Asia. Moreover, smoking, alcohol and regular physical activity may be both the consequences of accelerated burnout and confounders to interplay with high work demands and other occupational burnout contributors in a complex way. The extent to which these lifestyle attributes can interact with burnout remains poorly understood, and little is known of whether regularly exercising, non-smoking doctors are less prone to burnout. Therefore, the aim of this analysis was to assess burnout prevalence in doctors and nurses of a cardiological hospital in Almaty, Kazakhstan and ascertain whether smoking, alcohol and physical activity may predict job-associated burnout.

## Methods

### Study design and instruments

This cross-sectional study of the occupational burnout determinants in cardiologic medical personnel was carried out in the City Cardiological Centre of Almaty, the largest city of Kazakhstan with the population over 2 million. We collected data using self-administered questionnaire in September 2018 in the workplace. The City Cardiological Centre of Almaty is the tertiary level facility located in the city centre and has a capacity of 265 beds. The center deals with all cardiovascular emergencies in the city, performs all cardiosurgical interventions, including urgent catheter surgery in myocardial infarction, but offers conservative in-patient treatment as well. The Centre employees are qualified doctors, nurses and technical personnel.

We offered a structured questionnaire to the Centre staff in Russian, which comprised demographic information, sociodemographic data, lifestyle questions, Maslach Burnout Inventory (MBI) Human Services Survey for Medical Personnel and Fatigue Severity Scale (FSS). Thus, we asked about sex, age, work duration in the Centre, year of birth, current position, current marital status (single; married, divorced), and the highest attained education (high school; college; university; academic degree). Lifestyle section of the questionnaire verified smoking status, alcohol consumption and the level of physical activity. Self-reported smoking status was categorized into never-smoker; ex-smoker and current daily smoker via three questions (“Have you ever smoked more than 100 cigarettes in your lifetime?”; “Do you continue smoking currently?”; “Do you smoke daily?”), whereas daily smokers were asked about the number of smoked cigarettes per day and the overall smoking duration. The level of physical activity was verified with a question “Are you engaged in any regular physical activity off-work at least three times a week for at least 40 minutes?”. Current alcohol consumption level was ascertained with the question “Choose the answer, which most accurately characterizes your alcohol consumption”, whereas the answers were “Never”; “No more than once a week” and “At least once a week”.

MBI Human Services Survey for Medical Personnel is a 22-item questionnaire, in which the answers are self-graded from 0 (Never) to 6 (Every day), and the overall score for each of three dimensions is the sum of answers on selected questions. Emotional Exhaustion (EE) dimension is the sum of nine questions; Depersonalization (DP) is the sum of five questions, whereas the remaining Personal Accomplishment subscale is the sum of the remaining eight questions. Higher subscale score reflects higher level of burnout with regard to EE and DP. PA score in inversely correlated with the burnout, where higher score is indicative of lower burnout. We compared the actual scores between doctors, nurses and other staff, but additionally we categorized subjects into those with “low”, “average” and “high” burnout, when the corresponding scores were ≤ 16 (EE) ≤6 (DP) ≥39 (PA) for “low”; 17–26 (EE), 7–12 (DP), 32–38 (PA) for “average”; and ≥ 27 (EE), ≥13 (DP), ≤31 (PA) for “high”.

Fatigue was measured with 9-item Fatigue Severity Scale (FSS), rating each item from 1 to 7 and summing all answers to produce FSS score. FSS score below or equal to 36 was treated as no fatigue, as opposed to score above 36 when fatigue was present.

### Statistical analysis

The primary outcomes in this study were three dimensions of burnout, including EE, DP and PA, as continuous variables and compared in univariate analyses between occupational groups. We also categorized burnout dimensions into low, average and high categories, as proposed by MBI. For normally distributed data, we used t-test for univariate analyses, whereas Mann-Whitney test was the alternative method. All binary data comparisons were performed using 2*2 tables and χ^2^ test. In most groups, burnout scores were left-slewed and presented as medians with the corresponding interquartile ranges (IQR). As expected, PA scores were right-skewed, because higher burnout had a lower score. The null hypothesis in this analysis was no difference in burnout levels between doctors and nurses; we also hypothesized that smoking, alcohol and physical activity did not mitigate occupational burnout in our sample. To test this hypothesis, we performed comparisons of continuous burnout scores of EE, DP and PA between doctors, nurses and other occupations using non-parametric tests. We also wished to identify the predictors of high burnout in those three burnout dimensions, and for this we stratified the sample into those with high and low-to-average scores and performed bivariate analyses of selected variables, such as sex, age, work duration, etc. We used NCSS 12 (Utah, USA) for all calculations, and data are presented as either median with IQR or the mean with the standard deviation (SD). *P*-values below 0.05 were considered significant.

## Results

Female doctors and nurses were the majority in this sample of the cardiology hospital (82%). Fifty-seven percent of the staff had either university education or an academic degree (Table 1). On average, work duration was 10 years, whereas two of three dimensions of burnout fell into the “average” category, including EE and DP. The mean PA burnout score was low. We found significant differences in smoking prevalence, alcohol use and regular physical activity between men and women. Men had generally attained higher level of education, with higher prevalence of smoking and alcohol use. Significantly more men were engaged in regular physical activity. None of included three dimensions of burnout differed between male and female staff.

**Table 1:** Participants’ sociodemographic characteristics and burnout scores

	All N (%)	Men N (%)	Women N (%)
N (%)	259 (100)	47 (18)	212 (82)
Age, years	34 (14)	31.5 (8.3)	35 (12)
Work duration, years	10 (13.3)	6.5 (11)	10 (15)
Marital status			
Single	86 (33)	15 (32)	71 (33)
Married	147 (57)	28 (60)	119 (56)
Divorced	26 (10)	4 (8)	22 (11)
Education			
High school	28 (11)	0 (0)	28 (13)*
College	83 (32)	5 (11)	78 (37)*
University	120 (46)	31 (66)	89 (42)*
Academic degree	28 (11)	11 (23)	17 (8)*
Smoking			
Never smokers	204 (79)	19 (40)	183 (86)*
Ex-smokers	29 (11)	11 (23)	19 (9)*
Daily smokers	26 (10)	17 (37)	10 (5)*
Alcohol			
Never	173 (67)	21 (45)	152 (72)*
Once a week or less	78 (30)	22 (47)	56 (26)*
More than once a week	8 (3)	4 (8)	4 (2)*
Regular physical activity	89 (34)	30 (64)	59 (28)*
FSS	27 (22)	24.9 ± 10.6	28 (23)
Burnout dimensions			
Emotional exhaustion	18 (15)	15 (15)	19 (15)
Depersonalization	12 (9)	12 (8)	12 (9)
Personal accomplishment	40 (15)	41 (11)	40 (14)

Note: Age, work duration, FSS and three dimensions of burnout scores are presented as either means±standard deviation or median with the corresponding interquartile range (in brackets) depending on the distribution; FSS – Fatigue Severity Score; * - p < 0.05 compared to men from either χ^2^ or Mann-Whitney tests

High EE, DP and PA burnout scores were found in 32, 52 and 16% of doctors and 26, 45 and 32% nurses. In both doctors and nurses, the prevalence of high DP burnout scores was much greater compared to EE or PA. EE median score fell into the “average” category in doctors and nurses, whereas in other occupations EE was on average low. Table 2 shows there were no statistically significant differences in the median scores of EE or DP when doctors were compared to nurses, nurses to other and doctors to other. However, PA burnout was significantly greater in nurses compared to doctors. This table also demonstrates that the number of people with high or low categories of selected dimensions of burnout may have been smaller in “other” positions, but very small sample size of this group limited statistical power and thus statistical significance was not attained in those comparisons.

**Table 2:** Burnout dimensions comparison between doctors, nurses and other medical staff

	Doctors	Nurses	Other
N (%)	96 (37)	151 (58)	12 (5)
Emotional exhaustion, median (interquartile range)	19 (15.8)	18 (17)	12 (19.5)
High, N (%)	31 (32)	40 (26)	3 (25)
Average, N (%)	28 (29)	41 (27)	2 (17)
Low, N (%)	37 (39)	70 (47)	7 (58)
Depersonalization, mean ± standard deviation or median (interquartile range)	14.1 ± 6.4	12 (9)	12 (10.3)
High, N (%)	50 (52)	68 (45)	4 (33)
Average, N (%)	38 (40)	56 (37)	4 (33)
Low, N (%)	8 (8)	27 (18)	4 (33)
Personal accomplishment, median (interquartile range)	41 (9)	38 (17)*	38 (18)
High, N (%)	15 (16)	48 (32)	5 (42)
Average, N (%)	15 (16)	34 (22)	1 (8)
Low, N (%)	66 (69)	69 (46)	6 (50)

Note: *significant difference when compared to doctors using Mann-Whitney U-test

We than stratified all participants into high vs. low or average categories of all three burnout dimensions. The prevalence of high burnout score across three dimensions was different. High EE and PA scores were found in about 30% of the staff, whereas up to half of the sample exhibited high DP scores (Table 3). Neither age nor work duration were associated with greater burnout. Similarly, sex, marital status and even smoking or physical activity could not predict higher scores of burnout. However, alcohol use was found to have a positive association with lower PA scores. Higher fatigue levels, expressed as higher FSS scores, predicted higher burnout in EE and DP, but not in PA, where the association was reverse, but still statistically significant.

**Table 3:** Selected predictors of high burnout scores across three dimensions

	EE		DP		PA	
	Low	High	Low	High	Low	High
N (%)	185 (71)	74 (29)	134 (52)	125 (48)	191 (74)	68 (26)
Male sex, N (%)	37 (20)	10 (14)	25 (19)	22 (18)	37 (19)	10 (15)
Age, years, mean ± standard deviation or median (interquartile range)	35 (15)	34.8 ± 8.7	35 (14.5)	33 (12)	34 (13.5)	37.1 ± 10.9
Work duration, years, median (interquartile range)	10 (15)	10 (10.5)	10 (15)	10 (12)	10 (14.3)	10.5 (12.5)
Married, N (%)	110 (59)	36 (49)	78 (58)	68 (54)	110 (56)	36 (53)
Never smoking, N (%)	147 (79)	55 (74)	105 (78)	97 (78)	146 (76)	56 (82)
Never alcohol, N (%)	128 (69)	44 (59)	86 (64)	87 (70)	119 (62)	52 (76)*
Regular physical activity, N (%)	70 (38)	19 (26)	46 (34)	43 (34)	66 (35)	22 (32)
FSS, mean ± standard deviation or median (interquartile range)	22 (18)	35.3 ± 12,6*	20 (17.3)	33.1 ± 13,2*	28 (22)	21.5 (22.5)*

Note: *EE* emotional Exhaustion, *DP* Depersonalization, *PA* Personal Accomplishment; *significant difference using Mann-Whitney U-test for Fatigue Severity Score (FSS) or 2*2 test with the corresponding χ^2^

## Discussion

To our best knowledge, this is the first report from any of five Central Asian countries on the prevalence of occupational burnout in doctors and nurses. In this study of almost 260 employees of the tertiary level cardiology hospital, we found no differences in the EE and DP between doctors and nurses, but PA burnout was statistically significantly greater in nurses. Moreover, one-third of doctors and nurses reported high scores of EE burnout, whereas high DP was identified in almost 50% of the staff. Such outstandingly great prevalence of selected burnout scores, including DP, in the cardiology doctors and nurses may be indicative of higher demands with poor reward and clearly prompts directions for preventative interventions with a purpose to improve the efficiency of the hospital operation. Another important finding in this analysis was the association of alcohol use with lower burnout in PA.

Data on the occupational burnout in physicians are not consistent from various settings, and the understanding whether work duration or age can predict such burnout is lacking. In a recent report from a university general hospital [6], high DP was found in only about 20% of physicians and nurses, which contrasts with our results from cardiology staff. In that report they found that nurses were scored higher burnout in EE and PA, which was also the case with regard to PA in our sample. In many other studies researchers report high burnout scores in the majority of medical personnel, most seen in intensive care providers [8, 9], but there exists significant variability in the estimates of both the mean score and high score prevalence from the studies around the world. The latest systematic review [10] has demonstrated that all three dimensions vary from 0 to almost 90%, but the cut-off levels and the definitions across the studies are so different, that they hamper identification of burnout with age, sex and other predictors. Numerous studies in that systematic review focused on occupational workload and stress as predictors of burnout, and very few could assess the association of burnout with lifestyle attributes, such as physical activity [11]. The latter was found to have a protective effect against burnout, which we could not confirm in our sample.

In addition to occupational workload and stress, social support and work-family conflict may modify the effect of stress on burnout. Although the role of work-family conflict may be less pronounced than of job satisfaction itself [12], it has been shown to raise the odds of burnout up to 6-fold in the emergency physicians [13]. Studies with such positive association conclude the need to consider work-family conflict and even family composition as important determinants of burnout. Since family size can been seen as a moderator between burnout and recovery [14] and more kids in family are associated with slower burnout [15], families as part of more general term of social support can take some action in the mitigation of the occupational burnout in medicals. In other words, the issue of burnout spreads beyond the workplace only, and the potential of good and balanced family relations in controlling occupational burnout in families should be kept in mind.

The interaction of burnout with alcohol may be more complex. Some association of alcohol use and burnout may seem plausible and was found both in the general analysis and of selected burnout dimensions [16, 17]. In the study of Danish physicians [16], the greatest effect was found for DP compared to EE and PA. In contrast, other report could not identify greater likelihood of burnout in those with alcohol use of problems [18]. Our presentation could not support any of these, as we found that those using alcohol have exhibit lower PA burnout compared to never alcohol users. This is somewhat a surprising finding, but may prompt further examination to understand the underlying mechanism of such association.

We could not confirm age and work duration could affect burnout, which is consistent with few other reports [19]. Other studies on the interaction of age and burnout and using a more comprehensive analysis showed that older age was associated with lower work capacity when high levels of burnout were present with the reverse association in lower levels of burnout in nurses [20]. This is indeed what we can somehow confirm with the association of FSS score with burnout scores in our doctors and nurses. However, age and FSS seem not to have a direct relationship, because in our analyses age could not predict any of the burnout scores. Therefore, fatigue, but not age may be important in progressing burnout.

The limitations of this analysis originate from the study design and the sample size. The new findings emerged in the study, such as the association of alcohol use with the lower burnout score, cannot be explained in a cross-sectional study, but may help plan directions for future research. We believe that alcohol and burnout are not random variables and should be deeper explored for their complex interaction. Also, we cannot ascertain the temporality of smoking, alcohol or physical activity with burnout. Secondly, although we included as many people from the cardiology hospital to achieve greater statistical power, there were people whom we could not include due to organizational issues, such as sick or vacation leave. Thirdly, the study may not be universally compared to other reports of this similar design, since a series of various instruments to measure burnout are available, and the choice of a specific questionnaire, such as MBI, may not explain all the variability of burnout. Another limitation is the participation of one center only, whereas the range of burnout scores may be wide when non-central hospitals are included. Finally, in such predominantly female sample, small number of men may limit the power of comparisons of sex differences in burnout scores.

High DP scores in cardiologists may reflect overall growing disappointment in medicine and positive treatment outcomes and therefore may results in poorer healthcare outcomes. A range of preventative interventions should be reviewed by the hospital management, and we would recommend to reduce the overall doctors and nurses’ workload, optimize professional rewarding system, including salaries, and some motivational support, such as providing more opportunities for doctors to interact with their peer abroad at conferences and other meetings. Additionally, positive psychology now offers some potential to improve cardiologists’ well-being as both a professional and individual [21]. Resilience-based approach is gaining popularity in recent years, and such approach may include the combination of high self-esteem, a more positive way of explaining events and low perfectionism [22].

## Conclusions

In conclusion, this is the first presentation of occupational burnout from physicians in Central Asia to show very high rates of DP scores in both doctors and nurses of the cardiological hospital. Regular physical activity may not mitigate high burnout in medical professionals, therefore, prevention strategies should probably address work demands in the workplace.

## Abbreviations

DP: Depersonalization
EE: Emotional exhaustion
FSS: Fatigue severity scale
IQR: Interquartile range
MBI: Maslach burnout inventory
PA: Professional accomplishment

## References

[1] Maslach C, Jackson SE (1981). The measurement of experienced burnout. J Organ Behav.

[2] Kraft U (2006). Burned out. Sci Am Mind.

[3] Rothenberger DA (2017). Physician burnout and well-being: a systematic review and framework for action. Dis Colon Rectum.

[4] Shanafelt TD, Balch CM, Bechamps GJ, Russell T, Dyrbye L, Satele D (2009). Burnout and career satisfaction among American surgeons. Ann Surg.

[5] Shanafelt TD, Hasan O, Dyrbye LN, Sinsky C, Satele D, Sloan J, et al. Changes in burnout and satisfaction with work-life balance in physicians and the general US working population between 2011 and 2014. In: Mayo Clinic proceedings: Elsevier; 2015. p. 1600–13. 10.1016/j.mayocp.2015.08.023 26653297

[6] Marques MM, Alves E, Queirós C, Norton P, Henriques A (2018). The effect of profession on burnout in hospital staff. Occup Med.

[7] West CP, Dyrbye LN, Erwin PJ, Shanafelt TD (2016). Interventions to prevent and reduce physician burnout: a systematic review and meta-analysis. Lancet.

[8] See KC, Zhao MY, Nakataki E, Chittawatanarat K, Fang W-F, Faruq MO, et al. Professional burnout among physicians and nurses in Asian intensive care units: a multinational survey. Intensive Care Med. 2018:1–12. 10.1007/s00134-018-5432-1 30446797

[9] Rajan S, Engelbrecht A. A cross-sectional survey of burnout amongst doctors in a cohort of public sector emergency centres in Gauteng, South Africa. Afr J Emerg Med. 2018. 10.1016/j.afjem.2018.04.001 PMC6223592 30456156

[10] Rotenstein LS, Torre M, Ramos MA, Rosales RC, Guille C, Sen S (2018). Prevalence of burnout among physicians: a systematic review. JAMA..

[11] Olson SM, Odo NU, Duran AM, Pereira AG, Mandel JH (2014). Burnout and physical activity in Minnesota internal medicine resident physicians. J Grad Med Educ.

[12] Yang S, Liu D, Liu H, Zhang J, Duan Z (2017). Relationship of work-family conflict, self-reported social support and job satisfaction to burnout syndrome among medical workers in Southwest China: a cross-sectional study. PLoS One.

[13] Estryn-Behar M, Doppia MA, Guetarni K, Fry C, Machet G, Pelloux P (2011). Emergency physicians accumulate more stress factors than other physicians–results from the French SESMAT study. Emerg Med J.

[14] Ugwu FO, Ugwu C, Njemanze VC, Nwosu I. Family cohesion and family size moderating burnout and recovery connection. Occup Med. 2018. 10.1093/occmed/kqy155 30476256

[15] Chatani Y, Nomura K, Horie S, Takemoto K, Takeuchi M, Sasamori Y (2017). Effects of gaps in priorities between ideal and real lives on psychological burnout among academic faculty members at a medical university in Japan: a cross-sectional study. Environ Health Prev Med.

[16] Pedersen AF, Sørensen JK, Bruun NH, Christensen B, Vedsted P (2016). Risky alcohol use in Danish physicians: associated with alexithymia and burnout?. Drug Alcohol Depend.

[17] Rath KS, Huffman LB, Phillips GS, Carpenter KM, Fowler JM (2015). Burnout and associated factors among members of the Society of Gynecologic Oncology. Am J Obstet Gynecol.

[18] Hyman SA, Shotwell MS, Michaels DR, Han X, Card EB, Morse JL (2017). A survey evaluating burnout, health status, depression, reported alcohol and substance use, and social support of anesthesiologists. Anesth Analg.

[19] Goldberg R, Boss RW, Chan L, Goldberg J, Mallon WK, Moradzadeh D (1996). Burnout and its correlates in emergency physicians: four years’ experience with a wellness booth. Acad Emerg Med.

[20] Hatch DJ, Freude G, Martus P, Rose U, Müller G, Potter GG (2018). Age, burnout and physical and psychological work ability among nurses. Occup Med.

[21] Panagioti M, Geraghty K, Johnson J (2018). How to prevent burnout in cardiologists? A review of the current evidence, gaps, and future directions. Trends Cardiovasc Med.

[22] Johnson J, Panagioti M, Bass J, Ramsey L, Harrison R (2017). Resilience to emotional distress in response to failure, error or mistakes: a systematic review. Clin Psychol Rev.

